# Characterizing Mealtime Verbal Interactions Among Nursing Home Staff and Residents With Dementia

**DOI:** 10.1093/geroni/igaa057.1225

**Published:** 2020-12-16

**Authors:** Wen Liu, Kristine Williams, Melissa Batchelor, Yelena Perkhounkova, Maria Hein

**Affiliations:** 1 University of Iowa, Iowa City, Iowa, United States; 2 University of Kansas, Lawrence, Kansas, United States; 3 The George Washington University, Washington, DC, United States

## Abstract

Mealtime difficulties are common in residents with dementia, leading to negative outcomes. Interaction with staff are critical to engage residents in eating. This study characterized dyadic verbal interactions (descriptive statistics), and relationships among verbal behaviors and between verbal behaviors and individual characteristics (bivariate analyses). This secondary analysis of 110 videotaped mealtime observations involved 25 residents and 29 staff (42 unique dyads) in 9 nursing homes (NH). Verbal behaviors (utterances) were coded using the Cue Utilization and Engagement in Dementia mealtime video-coding scheme, addressing 8 positive behaviors and 4 negative behaviors. Staff spoke three times more frequently (76.5%) than residents (23.5%). Nearly all staff utterances were positive (99.2%). 85.1% of residents’ utterances were positive and 14.9% negative. Staff positive utterances were associated with staff negative utterances (p=.02), and resident positive (p<.001) and negative (p<.001) utterances. Staff negative utterances were associated with resident negative utterances (p=.02), but not with resident positive utterances (p=.39). Resident positive and negative utterances were associated (p<.001). Staff positive utterances were associated with staff race (p=.01), and resident age (p=.01), dementia stage (p<.001), and eating function (p<.001). Resident positive utterances were associated with years staff worked as caregivers (p=.02) and in the current NH (p=.01), resident age (p=.04), comorbidity (p=.04), dementia stage (p=.01), and eating function (p=.003). Resident negative utterances were associated with dementia stage (p=.01). Dyadic mealtime interactions were dynamic, interactive and complex. Multiple resident and staff characteristics were related to mealtime verbal interactions. Findings may inform development of individualized, person-centered mealtime care interventions.

